# Cytohesin-2/ARNO: A Novel Bridge Between Cell Migration and Immunoregulation in Synovial Fibroblasts

**DOI:** 10.3389/fimmu.2021.809896

**Published:** 2022-01-12

**Authors:** Yilin Wang, Çağlar Çil, Margaret M. Harnett, Miguel A. Pineda

**Affiliations:** ^1^ Institute of Infection, Immunity and Inflammation, University of Glasgow, Glasgow, United Kingdom; ^2^ Research Into Inflammatory Arthritis Centre Versus Arthritis (RACE), Glasgow, United Kingdom

**Keywords:** ARNO, synovial fibroblast (FLS), inflammation, arthritis (including rheumatoid arthritis), cytohesin 2

## Abstract

The guanine nucleotide exchange factor cytohesin-2 (ARNO) is a major activator of the small GTPase ARF6 that has been shown to play an important role(s) in cell adhesion, migration and cytoskeleton reorganization in various cell types and models of disease. Interestingly, dysregulated cell migration, in tandem with hyper-inflammatory responses, is one of the hallmarks associated with activated synovial fibroblasts (SFs) during chronic inflammatory joint diseases, like rheumatoid arthritis. The role of ARNO in this process has previously been unexplored but we hypothesized that the pro-inflammatory milieu of inflamed joints locally induces activation of ARNO-mediated pathways in SFs, promoting an invasive cell phenotype that ultimately leads to bone and cartilage damage. Thus, we used small interference RNA to investigate the impact of ARNO on the pathological migration and inflammatory responses of murine SFs, revealing a fully functional ARNO-ARF6 pathway which can be rapidly activated by IL-1β. Such signalling promotes cell migration and formation of focal adhesions. Unexpectedly, ARNO was also shown to modulate SF-inflammatory responses, dictating their precise cytokine and chemokine expression profile. Our results uncover a novel role for ARNO in SF-dependent inflammation, that potentially links pathogenic migration with initiation of local joint inflammation, offering new approaches for targeting the fibroblast compartment in chronic arthritis and joint disease.

## Introduction

Fibroblasts are stromal cells of mesenchymal origin that, in addition to their well-described structural roles, are essential for maintaining tissue homeostasis, as they orchestrate local immunity and inflammation, promote wound healing and control matrix remodeling (1). Thus, dysregulation of fibroblast activity by genetic, epigenetic or even environmental factors can prime pathological networks leading to chronic disease and autoimmunity (2). Such pathogenic fibroblast activation has been demonstrated in inflammatory Rheumatoid Arthritis (RA), a chronic inflammatory disease affecting the joints.

In healthy conditions, fibroblasts are essential to provide nutritional support and preserve joint homeostasis. Thus, fibroblasts cooperate with tissue-resident macrophages to form the synovium, a unique membranous organ lining the joint cavity whose barrier function maintains immune privilege in the joint (3). However, during RA, synovial fibroblasts (SFs) undergo a pathogenic transformation that destabilizes the normal function of the synovium and triggers inflammatory mechanisms, such as recruitment of immune cells, production of inflammatory cytokines (IL-6, CCL2, MCSF, RANKL), formation of ectopic lymphoid-like structures and responses to inflammatory factors (IL-1β, TNF, IL-17) (4–6). As a result, inflammatory SFs establish self-perpetuating inflammatory circuits based on secreted factors. Additionally, SFs-dependent pathogenesis is associated with changes in their adhesion and migration capacities, which ultimately creates the pannus, an overgrowth of the synovium observed only in arthritic joints that invades and damages bone and cartilage. As also observed in tumorogenesis, pathological SF migration is a result of dysregulated expression of matrix degrading enzymes, like MMPs, and aberrant upregulation of adhesion molecules, such as cadherin-11, CD82 and integrins or focal adhesion kinase (FAK) (7–9). Although the characterisation of functional heterogeneity provided by single-cell RNA-Sequencing (RNA-Seq) has allowed a rapid advance in the understanding of the pathophysiology of SFs in RA (1, 10–12), the mechanisms that underpin SFs migration and invasiveness are still unclear. This could be because Next Generation Sequencing (NGS) does not consider the influence of the extracellular matrix and/or post-translational regulatory mechanisms governing cell signalling, the impact of which on cell function would not be detected by transcriptomic approaches alone.

Relating to these cell communication aspects, ADP-ribosylation factors (ARFs) are a family of small GTPases with roles in modulating membrane structure and the intracellular vesicle transport required for cell motility and attachment of cells to matrix components (13–16). ARF function is post-translationally regulated by guanine nucleotide exchange factors (GEFs), exchanging GDP for GTP to generate the active GTP-ARF. One particular GEF, cytohesin-2 (Cyth-2), also known as ARF nucleotide-binding site opener (ARNO), has been shown to promote cellular adhesion and migration in several cell types. ARNO-dependent activation of ARF6 induces recycling of integrin β1 (17) and modulates actin remodeling (18, 19) even in the absence of its GEF activity (20). Interestingly, Zhu et al. (21) also showed that ARNO acts as a branch point, separating IL-1β-dependent effects on cytokine secretion and barrier permeability in endothelial cells. Specifically, IL-1β triggers the MyD88-ARNO-ARF6 axis to compromise the integrity of the endothelial biological barrier, diverging from the canonical IL-1β-MyD88-IKK-NFκB pathway that promotes cytokine secretion and inflammation. Exposure of mice undergoing Collagen-Induced Arthritis (CIA) to the cytohesin inhibitor SecinH3 reduced disease severity and vascular permeability in the joints (21), but regulatory effects of ARNO on SFs in an inflammatory environment cannot be ruled out.

We thus hypothesized that inflammatory cytokines in the arthritic joint upregulate expression of ARNO in SFs, in turn activating ARF6-dependent pathways that could promote the invasive phenotype characteristic of arthritic SFs. Thus, ARNO could offer a novel molecular switch discriminating between inflammatory and invasive fibroblasts in the arthritic synovium. Consistent with this hypothesis, we observed that ARNO was up-regulated upon IL-1β stimulation in SFs *ex vivo*. Likewise, ARNO was found to be necessary to induce the morphological changes required for SFs migration. However, in contrast to the responses reported for endothelial cells (21), ARNO is also involved in regulation of SF inflammatory cytokine secretion. Collectively, our results indicate that the role of ARNO as a molecular checkpoint between inflammation and disruption of barrier permeability is not a universal function of this regulatory protein but rather depends on the cell type and anatomical location.

## Methods

### *Ex Vivo* Culture of SFs

Murine SFs were expanded *ex vivo* as described previously (22). Briefly, joint tissue was dissected from mouse paws and subjected to collagenase IV digestion (1 mg/ml) for 80 minutes at 37°C in DMEM. Cells were then cultured for 24 h to allow attachment to flasks and subsequently expanded in DMEM supplemented with 10% fetal calf serum (FCS), 1% penicillin/streptomycin, 1% glutamine and 1% non-essential amino acid (all Invitrogen, UK) in 5% CO2 at 37°C. Any contaminating myeloid cells were removed from cultures prior to experimental setup by negative selection using a biotinylated anti-CD11b antibody (Biolegend, #101204) followed by subsequent Streptavidin MicroBeads (Miltenyi Biotec, UK) for magnetic separation. Purity of SFs cultures (>99.5%) was assessed by positive expression of podoplanin by Flow Cytometry using anti-podoplanin-Alexa Fluor 647 (#156204, Biolegend, UK) and anti-CD11b-FITC (#11-0112-85, Invitrogen, UK). For *in vitro* stimulation of SFs, recombinant IL-1β, TNFα and IL-17 (Immunotools, Germany) were used for the indicated times at 10 ng/ml. STAT3 activation was inhibited *in vitro* using Cpd188 (Sigma-Aldrich, UK) for 30 minutes at 73 µM.

### Short Interference RNA (siRNA)

ARNO siRNA (Mm_Pscd2_3) or a negative non-specific siRNA control (Allstars siRNA) were transfected into SFs using HiPerFect transfection reagent (all Qiagen, UK) according to the manufacturer’s protocol. Briefly, siRNAs were diluted in 8% HiPerFect Transfection Reagent in DMEM (Invitrogen, UK) and incubated for 10-15 minutes at room temperature to form transfection complexes before adding to the cells (final siRNA concentration 10 nM). Cells were incubated with transfection complex for 24 hours and then washed with complete DMEM. Cells were transfected again using this protocol after 3 days, prior to experimental analysis as indicated.

### Collagen-Induced Arthritis (CIA) Mouse Model

Mice (8-10 weeks old male DBA/1 mice) were obtained from Envigo (UK) and maintained in the Biological Service Facility at University of Glasgow in line with the guideline of Home Office UK and AWERB, the ethics review board of the University, and licenses PIL IF5AC4409, PPL P8C60C865. Mice were immunized with type II bovine collagen (100 μg, MD Biosciences) emulsified in complete Freund’s adjuvant (CFA, day 0), and then challenged with type II bovine collagen (200 μg, in PBS) on day 21. Body weight, clinical scores and paw thickness were measured every two days. Disease scores were measured in a scale from 0-4 for each paw, 0 = no disease, 4 = very inflamed/loss of function. An overall score of 10 or more was considered an experimental endpoint and animals were immediately euthanized. Naive mice; weight: 26.96 ± 1.65 g, paw width: 1.82 ± 0.07 mm. CIA mice body; weight: 23.96 ± 2.45 g, paw width: 2.225 ± 0.22 mm, clinical scores: 6.1 ± 3.62.

### RNA Isolation and qRT-PCR

RNA from SFs was extracted using either EZ-10 RNA Mini-Preps Kit (Bio Basic, USA) or RNeasy Micro Kit (Qiagen, UK) according to manufactures’ instructions. cDNA was synthesized with High-Capacity cDNA Reverse Transcription Kit (Thermo Fisher Scientific, UK). Gene expression was measured by RT-qPCR which was performed using either KiCqStart^®^ SYBR^®^ Green Primers (sigma-aldrich, UK) or TaqMan™ Gene Expression Assay (Thermofisher, UK) as indicated. Actin was used as an endogenous control to normalise samples and relative gene expression was evaluated using the comparative C_t_ (ΔΔC_t_) method. Sybr green primers used were actin/NM_007393, Cxcl9/NM_008599, Ccl9/NM_011338 and Cldn1/NM_016674 whilst Taqman mRNA primers were used for Actb (Mm02619580_g1), ARNO (Mm00441008_m1), IL-6 (Mm00446190_m1), CCL2 (Mm00441242_m1), MMP3 (Mm00440295_m1), MMP13 (Mm00439491_m1), TNFRSF11b (Mm00435454_m1) and TNFSF11 (Mm0041906_m1).

### Immunofluorescence Staining

Cells seeded on chamber slide were incubated in Fixation buffer (Biolegend, UK) for 10 min at room temperature, washed three times with PBS, permeabilised (0.5% Triton X-100 in PBS) for 10 min and treated with blocking buffer (PBS 1% BSA or 10% normal serum from the species in which the secondary was raised) prior to incubation with primary antibodies overnight at 4°C. The following primary antibodies were used: anti-vinculin (V9264, Sigma, UK) and Alexa Fluor™ 488 Phalloidin (A12379, Invitrogen, UK). Samples were then incubated with donkey anti-mouse Alexa fluor 647 (A31571, Invitrogen, UK) for 1h at RT. Slides were mounted with SlowFade™ Diamond Antifade Mountant with DAPI (S36968, Invitrogen, UK) to counterstain nuclei. Samples were visualized using an EVOS (EVOS™ FL Auto 2, Thermofisher, UK) microscope.

### ARF6-GTP Pull Down Assay

Activation of ARF6 (ARF6-GTP) was quantitated using the Arf6 Pull-Down Activation Assay Biochem Kit from Cytoskeleton (BK033-S, USA) according to manufacturer’s instructions. Briefly, cells were lysed on ice in 200 μl Cell Lysis buffer and centrifuged at 10,000 x g for 1 min at 4°C. An aliquot (20 μg) of lysate was saved for Western blot quantitation of total ARF6 expression. Then, cell lysate (125 μg) was added to 5 μg GGA3-PBD beads (Cytoskeleton, USA) to pull down ARFs-GTP and incubated at 4°C on a rotator for 1h and then the beads were extensively washed to remove unbound protein. The beads were then resuspended and boiled in Laemmli buffer (SigmaAldrich, UK) and the samples resolved by SDS-PAGE prior to detection of ARF6 by Western blot.

### Western Blotting

Cells were harvested and lysed in RIPA buffer (Thermo Fisher Scientific, UK) containing protease inhibitor and PhosSTOP tablets (Roche, UK) and sample protein concentration determined using the Pierce^®^ BCA Protein Assay Kit (Thermo scientific, UK). SDS-PAGE was then performed, and proteins (20-30 μg) were transferred onto nitrocellulose membranes. Membranes were blocked with TBS-T 5% non-fat milk prior to overnight incubation with for primary antibodies at 4°C (used at 1:5000 unless stated otherwise). The following antibodies were used: anti-Cytohesin2 (sc-374640; Santa Cruz Biotechnology, 1:1000), anti-ARF6 (ARF-06; Cytoskeleton, 1:500), anti-GAPDH (CST #2118, UK), anti-p38 (CST #9212, UK), anti-p38 phospho (CST #9211, UK), anti-STAT3 (CST #9139, UK) and anti-STAT3 phospho (CST #9145, UK), anti-ERK1/2 phospho (CST #9101, UK), anti-ERK1/2 (CST #4695, UK), anti-c-Jun phospho (CST #9261, UK, 1:1000) and anti-c-Jun (CST #9165, UK). Membranes were then incubated with anti-rabbit (CST #7074P2, UK) or anti-mouse (CST #7076s, UK) anti-IgHRP-conjugated secondary antibodies for 1h at room temperature and washed three times in TBS-T. Expression signal was detected by ECL Western Blotting Substrate (Thermo scientific, UK) and the protein bands were quantified using GelAnalyzer 2010a software with the relative integrated density values normalised to ERK1/2 and GAPDH expression values.

### Cytokine Detection by ELISA

Cell supernatants were collected for evaluation of secreted cytokines. SFs (10^4^) were seeded in 200 μl of complete DMEM in 96-well plates pre-coated with bovine fibronectin (R&D, UK). For siRNA silencing experiments, the first siRNA transfection was performed in 12-well plates and when the first transfection was completed, the cells were detached for a second transfection and re-seeded in 96-well plates. Cells were cultured in the absence or presence of 10 ng/ml IL-1*β* for 24 hours, then supernatants were collected and frozen. ELISA kits (Dou set, R&D) were used to evaluate the levels of IL-6, MMP3 and CCL2 in the supernatants according to the manufacturer’s instructions. Cell viability was checked using a colorimetric MTS assay (abcam, catalog number ab223881).

### Cell Migration Assay

4-well µ-Dishes (#80466, Ibidi, UK) coated with fibronectin were seeded with 10,000 SFs for all the experimental groups. Dishes had 2 distinct culture spaces delineated by a silicon insert. Cells were grown until confluency, and inserts were then removed. Removal of the inserts left an empty space in the wells, where cells grew to fill the gap. The space without cells right after insert removal was measured as a reference for subsequent cell migration, and it was measured again after 24 hours. Images were acquired using an EVOS microscope EVOS™ FL Auto 2, Thermofisher, UK and the distance migrated was calculated using ImageJ software.

### Flow Cytometry

The following antibodies were used with flow cytometry: anti-Podoplanin-Alexa Fluor 647 (#156204, Biolegend, UK), anti-CD11b-FITC (#11-0112-85, Invitrogen, UK), DAPI (#32670, Sigma, UK). Cells were trypsinized, stained at 4°C in PBS containing 2mM EDTA and 0.5% FCS and analysed using a LSR II flow cytometer (BD Biosciences). For proliferation studies, synovial fibroblasts were labelled with 10 μM proliferation dye eFluor 670 (#65-0840-90, eBioscience, UK) for 10 min on ice. Labelling was stopped by adding 5 volumes of DMEM 10% FCS culture medium and incubated 5 minutes on ice. DAPI was used to discriminate live and dead cells. Data were analyzed with FlowJo software 10.7.1.

### RNAseq and Bioinformatic Analysis

Total RNA from cultured synovial fibroblasts was isolated using RNeasy Micro kit (Qiagen, Germany). RNA integrity was checked using the Agilent 2100 Bioanalyzer System, and the RIN value was >9 for all samples. Library preparation was done using the TruSeq mRNA stranded library preparation method and samples were sequenced (2 × 75 bp) with an average of 30 million reads. All RNA-seq reads were then aligned to the mouse reference genome (GRCM38) using Hisat2 version 2.1.0, and read counts were generated using Featurecounts version 1.4.6. Data quality control, non-expressed gene filtering, median ratio normalization (MRN) implemented in DESeq2 package, and identification of differentially expressed (DE) genes (padj < 0.01, |foldChange|>2) was done using the R Bioconductor project DEbrowser (23). Gene Ontology (GO) enrichment and KEGG pathway enrichment was conducted with Metascape (24). [10/12/2021 15:40]. ClustImpute package under the R software was used to identify clusters among naive, and IL-1β stimulated cells, control and ARNO siRNA treated.

### Statistical Analysis

All statistical analysis was performed in GraphPad Prism 8. Data are presented as the mean ± standard error (SEM) with students t-test used to show differences between two study groups for unpaired samples. P values <0.05 were considered significant.

## Results

### IL-1β Activates the ARNO-ARF6 Pathway in Murine SFs

**Figure 1:**
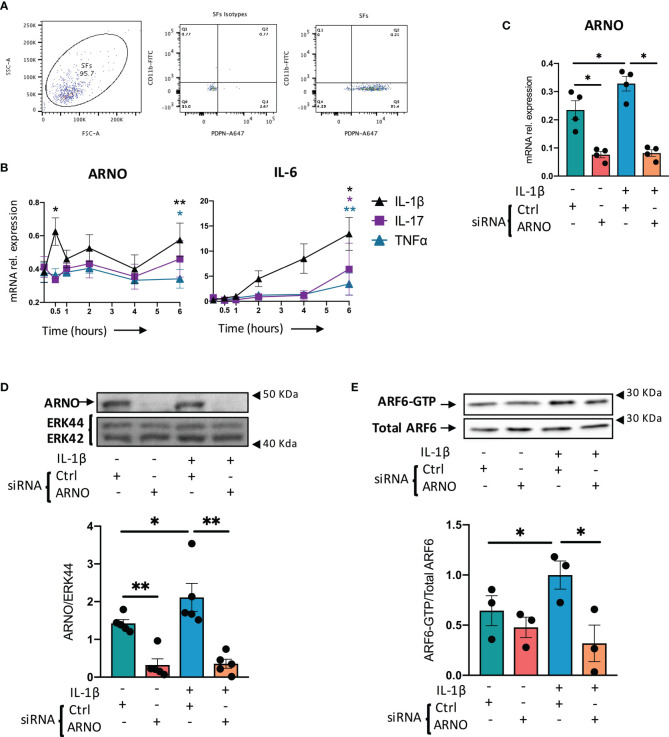
IL-1β upregulates ARNO in SFs. **(A)** SFs were isolated from mouse synovium and expanded *ex vivo*. Cells were trypsinased and expression of podoplanin (PDPN) and CD11b was evaluated by flow cytometry. **(B)** SFs were stimulated with IL-1β, IL-17 and TNFα (10 ng/ml) for 30 minutes and 1, 2, 4 and 6 hours, when RNA was extracted. Expression of ARNO and IL-6 mRNA was then quantified by RT-qPCR. Data show the mean of at least three independent experiments (analysed in technical triplicates) ± SEM. **(C)** Naïve SFs were transfected with either ARNO or control Allstars siRNA, followed by stimulation with recombinant IL-1β (10 ng/ml) when indicated. RNA was extracted after 6 hours and ARNO mRNA expression was evaluated by RT-qPCR. Each dot represents an independent experiment (analysed in technical triplicate), and error bars represent SEM. **(D)** SFs were transfected and stimulated as in **(C)**, and proteins were extracted 24h after stimulation. ARNO protein was detected by specific monoclonal antibodies by Western bloting and total Erk was used as loading control. Image shows one representative experiment. Column graph shows quantification of band intensity for ARNO normalised to Erk44. **(E)** SFs treated with Allstars or ARNO siRNA followed by IL-1β stimulation (5 minutes) were assayed by ARF6-GTP pull down assay and subsequently immunoblotted with anti-ARF6 antibody. Relative activation of ARF6 was normalised to unstimulated control siRNA-treated samples. For **(D, E)**, each dot represents an independent experiment and error bars represent SEM. *p < 0.05, **p < 0.01 by Wilcoxon’s matched-paired signed rank test in **(B)**, by Mann-Whitney test in **(C–E)**.

Synovial fibroblasts (SFs) were expanded from joint tissue collected from healthy animals as described previously (22) and their phenotype confirmed by their expression of podoplanin and the absence of the myeloid marker, CD11b (**Figure 1A**). To test our working hypothesis, we first evaluated expression of ARNO mRNA in SFs, both under resting conditions and upon stimulation with recombinant versions of pro-inflammatory cytokines typically found in the inflamed joint (**Figure 1B**). IL-1β, IL- 17 and TNF were selected as they have been shown to be drivers of disease both in animal models (25, 26) and in human RA, and have been associated with distinct pathotypes in RA (27). However, whilst all three cytokines upregulated IL-6 mRNA levels, only IL-1β significantly increased ARNO mRNA levels (**Figure 1B**) suggesting, in line with previous observations in endothelial cells (21), that IL-1β provides a specific signal(s) that promotes ARNO activity.

To address the functional role of ARNO in SFs, we used small interfering RNA (siRNA) approaches which were highly effective at downregulating expression of both ARNO mRNA (78.3% ± 0.08, **Figure 1C**) and ARNO protein (79.1% ± 0.22, **Figure 1D**), relative to the negative control AllStars siRNA (Qiagen, siRNA with no homology to any known mammalian gene) in resting and IL-1β-stimulated SFs. ARNO activates ARF6 to compromise vascular permeability in arthritis and other inflammatory conditions (21, 28, 29) and it also triggers key effector mechanisms involved in cell migration (29–32). Thus, to investigate whether the ARNO-ARF6 axis was also active in SFs, we knocked-down ARNO expression by siRNA and evaluated activation of ARF6 (GTP-bound) in response to IL-1β (**Figure 1E**). This showed that ARNO is necessary for IL-1β to induce ARF6 activation, confirming the presence of the IL-1β-ARNO-ARF6 axis in SFs.

### ARNO Is Necessary for SF Migration and Focal Adhesion Formation

**Figure 2:**
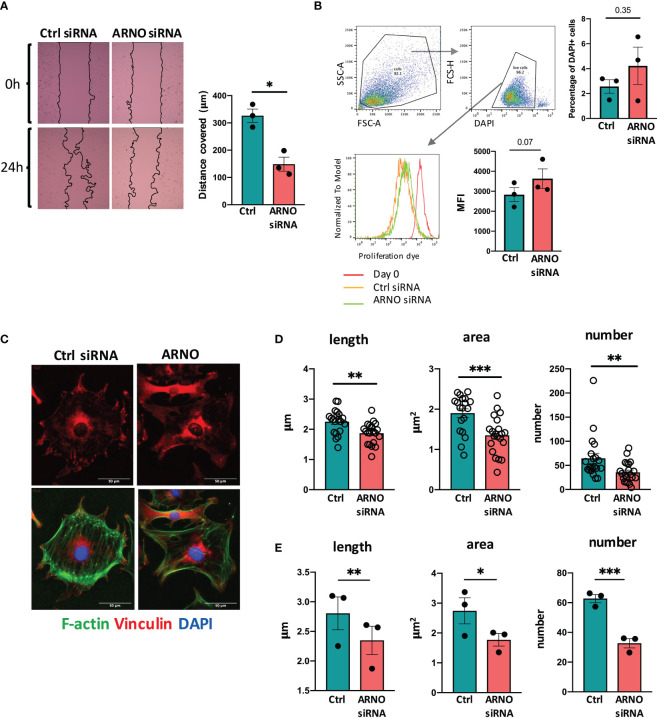
ARNO regulates SFs migration and assembly of focal adhesions. **(A)** SFs were seeded in migration chambers and grown until monolayer confluence. Cells were then treated with either Allstars control or ARNO siRNAs, when inserts were removed to perform migration assays. Pictures show one representative experiment, superimposed black lines delineate the cell-free area. Bar chart shows the mean of cell migration distance ± SEM from three independent experiments calculated with ImageJ software. **(B)** SFs were labelled with proliferation dye eFluor 670 (eBioscience), analysed by flow cytometry (day 0) or treated with control or ARNO siRNA, when cells were maintained in culture. After 5 days, mean fluorescence intensity was evaluated in live cells (identified by low DAPI staining). Histogram shows one representative experiment. Mean fluorescence intensity (MFI) of three independent experiments is shown in the column graph, error bars represent ± SEM. **(C)** Representative immunofluorescence staining of Vinculin (red) and F-actin (green) in SFs treated with Allstars control or ARNO siRNA. Scale bar: 50um. **(D)** Number, length and area of vinculin positive areas were analysed with ImageJ in SFs treated with Allstars control or ARNO siRNA. Each dot represents one single cell, data are from one representative experiment. **(E)** Mean of number, length and area of vinculin positive areas from three independent experiments, in SFs treated with Allstars control or ARNO siRNA. Error bars error bars represent ± SEM. *p < 0.05, **p < 0.01, ***p < 0.001 by Mann-Whitney test.

Next, we sought to characterise the physiological function(s) of ARNO in SFs. ARNO-ARF6 signaling promotes cell migration, through well-described mechanisms involving Rac1, DOCK180/Elmo and R-Ras and cytoskeleton-dependent changes in cell morphology (30, 33). Increased ARNO activity also modifies the way that cells interact and move through the extracellular matrix, by controlling β1-integrin recycling (33, 34). Because migration and integrin-dependent signaling are known hallmarks of inflammatory SFs, we evaluated the ability of SFs to migrate using wound healing assays (**Figure 2A**). SFs migration was significantly reduced when ARNO was knocked-down, in agreement with the function of ARNO described in other cell types. We also evaluated the effect on cell proliferation (dye) and cell death (DAPI high), but ARNO inhibition did not have a significant impact on either of those parameters (**Figure 2B**), suggesting that distinct molecular mechanisms are likely responsible for dysregulation of cell migration upon ARNO knock-down. To initiate migration, SFs in RA must disassemble cell contacts in the organized synovium and establish new ones with the extracellular matrix. We hypothesized that ARNO was involved in the formation of focal adhesions (FAs), multiprotein complexes that concentrate actin polymerization to control migration through engagement with the matrix. To investigate this, we stained SFs for vinculin (**Figure 2C**), a protein that anchors actin to the membrane in FAs. We found that ARNO siRNA significantly reduced both the number and structure (length and area) of focal adhesions (**Figures 2D, E**). Overall, these data highlight the role that ARNO plays in determining the fundamental biology of SFs, including their morphology, cell adhesion and migration.

### ARNO Is Required for Cytokine Secretion in Activated SFs

**Figure 3:**
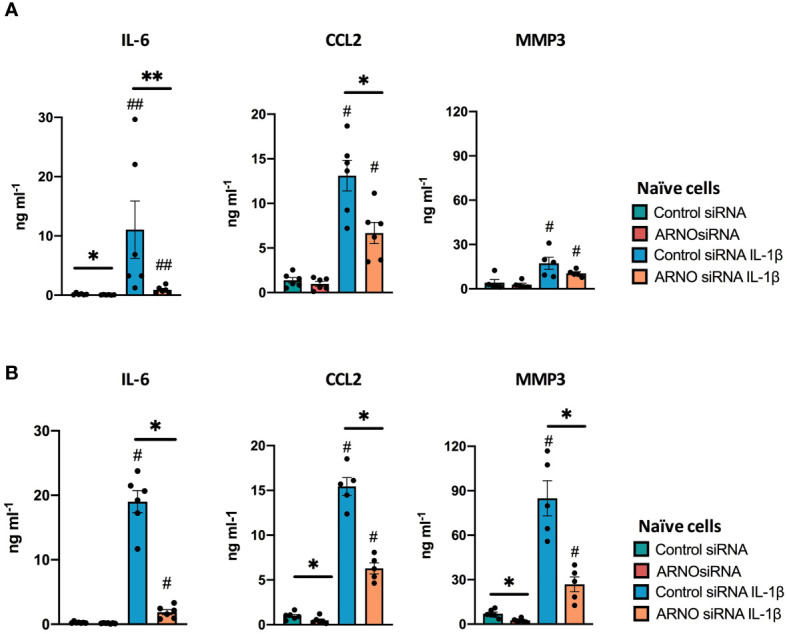
Reduction of ARNO expression down-regulates IL-1β-mediated cytokine expression. Secretion of IL-6, CCL2 and MMP3 was analysed by ELISA in the supernatant of control and ARNO siRNA treated naïve SFs **(A)** and SFs expanded from mice undergoing experimental Collagen-Induced Arthritis **(B)**. Cells were stimulated with IL-1β as indicated, and cell supernatants were collected after 24h for cytokine quantification. Each dot represents one independent experiment analysed in triplicate, error bars represent SEM. *p < 0.05, **p < 0.01 versus respective siRNA control; ^#^p < 0.05, ^##^p < 0.01 versus unstimulated control, Statistical significance was evaluated by the Mann-Whitney test.

Initiation of SF pathological migration is likely to be directly linked with inflammation because interactions with the matrix through up-regulated cell-to-cell adhesion molecules can increase secretion of cytokines and matrix metalloproteinases (MMPs) (35–37). Thus, although ARNO is not often reported as an inflammatory mediator, we proposed that formation of ARNO-dependent complexes upon IL-1β stimulation triggers secretion of inflammatory cytokines, coupling pathological invasiveness with local SF-derived inflammation. Consistent with this, we observed that reduction in ARNO expression (using siRNA) substantially inhibited IL-1β-dependent IL-6 and CCL2 secretion by naïve SFs (**Figure 3A**). Down-regulation of MMP3 was also observed, albeit this did not reach statistical significance (**Figure 3A**). To assess the prospect of using ARNO as a therapeutic target, it was important to establish whether the responses observed with naïve SFs also pertained in the chronically activated cells generated during disease. Thus, SFs explant cultures from animals undergoing experimental arthritis were examined for ARNO-dependent expression of IL-6, CCL2 and MMP3 (**Figure 3B**). We chose the Collagen-Induced Arthritis (CIA) model, as it shares hallmarks of human disease relevant for this study, such as the SF activation. As expected from previous studies (38), CIA SFs showed elevated cytokine production compared to naïve cells, corroborating their inflammatory phenotype. ARNO knock-down in such CIA cells reduced the IL-1β-dependent release of IL-6, CCL2 and MMP3, the latter also in a significant manner in these *in vivo* activated cells. Collectively therefore, we observed that suppression of ARNO limited the ability of both naïve and CIA SFs to respond to IL-1β, indicating that ARNO-signalling may provide a novel link between inflammation and matrix remodeling/invasiveness in SFs.

**Figure 4:**
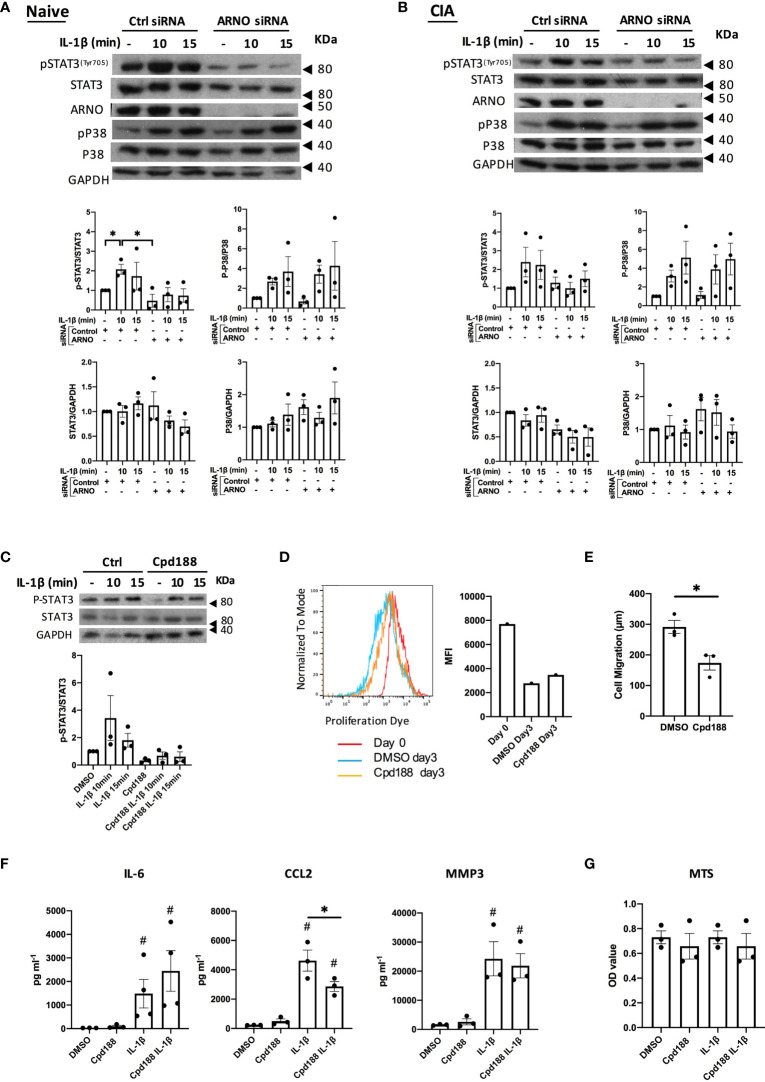
ARNO is required for STAT3 phosphorylation in SFs. Representative Western blots of the naïve **(A)** and CIA **(B)** SFs treated with control and ARNO siRNA followed by IL-1β stimulation (10 ng/ml) at indicated times. Anti-pSTAT3^Tyr705^, STAT3, ARNO, p-p38, p38 and GAPDH antibodies were used. Graphs show the relative quantification of phosphorylated STAT3 and p38 in naïve SFs and CIA SFs, calculated as pSTAT3/STAT3 and p-p38/p38 band intensity, and changes in total protein expression were calculated as STAT3/GAPDH and p38/GAPDH. Error bars represent SEM (n=3). Each dot represents one independent experiment. *p < 0.05 by Mann-Whitney test. **(C)** Representative Western blots and quantification of phosphorylated and total STAT3 in control (DMSO) and Cpd188 (STAT3 inhibitor, 73uM) treated naïve SFs followed by IL-1β stimulation at indicated times (n=3). **(D)** Mean fluorescence intensity of proliferation dye in SFs cultured for 3 days after treated with Cpd188 compared to control SFs. **(E)** Migration distance of SFs was measured 24 hours after Cpd188 treatment, error bars represent SEM (n=3). *p < 0.05. **(F)** Control or Cpd188-treated SFs were stimulated by IL-1β (24h), when cell supernatants were collected to analyse cytokine secretion by ELISA. ^#^p < 0.05 versus unstimulated control, statistical significance was evaluated by the Mann-Whitney test. **(G)** cell viability was assessed using MTS cell proliferation kit. In all cases, one dot represents one individual experiment, analysed in triplicate for ELISA and MTS assays.

To obtain further mechanistic insight, we investigated potential signaling pathways regulated by ARNO (**Figure 4**). Thus, we explored the role of ARNO in the IL-1β STAT3 signalling pathway, as this can play key roles in both acute and chronic inflammatory responses upon IL-1β stimulation in SFs (38). We also evaluated the p38 MAPK pathway, as it has been shown to be important for RA pathogenesis and IL-1β signalling (39) and also for ARNO signaling in relation to MMP expression (31). Reduction in ARNO expression by siRNA transfection resulted in inhibition of STAT3 activation (as evidenced by its phosphorylation and quantitated as pSTAT3/STAT3 expression) upon IL-1β stimulation, both in naïve (**Figure 4A**) and CIA SFs (**Figure 4B**). Inhibition of ARNO also seemed to induce some downregulation of total STAT3 expression, although the difference did not achieve statistical significance. By contrast, no effect was observed on p38 activation or expression, ruling out a direct role for this kinase in ARNO-mediated inflammation and for ARNO in p38 coupling. Likewise, we did not observe any effects on activation of the ERK and JNK (as evidenced by phosphorylation of c-Jun) MAPK pathways (**Supplementary Figure 1**), further supporting the importance of STAT3 in ARNO signalling. To provide further support for a functional role for the IL-1β-ARNO-STAT3 axis in SFs pathogenesis, we treated SFs with the selective STAT3 inhibitor Cpd188 (40), which efficiently blocked IL-1β-dependent activation of STAT3 (**Figure 4C**). Cpd188 treatment did not affect SF proliferation (**Figure 4D**), but it reduced migration in wound repair assays (**Figure 4E**), findings resembling the effects observed with ARNO knock-down (**Figures 2A, B**). Finally, we measured the effect of STAT3 inhibition on cytokine secretion upon IL-1β stimulation. (**Figure 4F**). In this case, results did not fully recapitulate those observed in ARNO knock-down experiments (**Figure 3**). Thus, although STAT3 inhibition down-regulated CCL2 release, the levels of IL-6 and MMP3 remained unaltered (**Figure 4F**), suggesting that additional ARNO-dependent signals might contribute to the production of these mediators. Cell viability was not affected by IL-1β/Cpd188 treatment (**Figure 4G**).

### ARNO-Signaling Fine-Tunes SF-Dependent Inflammation Upon IL-1β Stimulation

**Figure 5:**
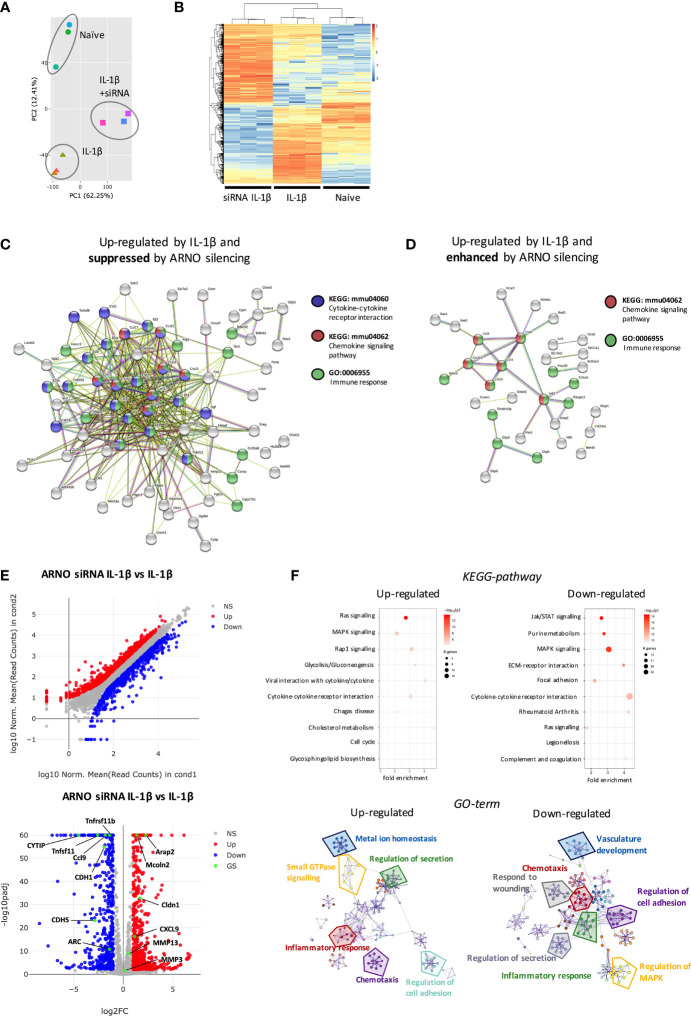
ARNO knocked-down SFs show a distinct transcriptomic profile in response to IL-1β stimulation. **(A)** RNA was isolated (RIN>9) from naïve, IL-1β stimulated and IL-1β stimulated ARNO knocked down SFs (6 hours, n=3) and subjected to bulk RNA-Seq (75bp paired-end, 30M reads). Principal component analysis (PCA) is shown. **(B)** Differential expression (DE) of genes amongst the three experimental groups. Genes were considered significant if they passed a threshold of padj < 0.01 and |log2foldChange| > 4 among any paired comparison. DE genes were then subjected to unsupervised hierarchical clustering and represented as Z scores. **(C,D)** Function enrichment and network analysis regulated by IL-1β and ARNO expression. STRING protein-protein interaction network (https://string-db.org) was performed on DE genes from **(B)**, with genes up-regulated only in the IL-1β-treated group **(C)** or genes up-regulated only in the ARNO siRNA group **(D)**. Significantly modulated pathways components associated with ARNO silencing upon IL-1β are shown. **(E)** DE of genes in IL-1β stimulated SFs compared to ARNO siRNA IL-1β SFs. Genes are plotted as a scatter plot where x= gene expression in IL-1β SFs, y=gene expression in siRNA ARNO IL-1β SFs. Volcano plot shows gene expression versus p value for each gene. Genes that pass a threshold of padj < 0.01 and |foldChange| > 2 in DE analysis are colored in blue when they are down regulated and red when they are upregulated in IL-1β treated cells. **(F)** DE genes identified in **(E)** were used to conduct KEGG pathway enrichment analysis and GO-term pathway analysis. KEGG pathways are represented by circles and are plotted according to fold enrichment on the x-axis and -log10 p-value on the coloured scale. The size is proportional to the number of DE genes. Metascape enrichment network diagrams illustrate GO-term pathways significantly enriched for top up-regulated and down-regulated genes.

To address identifying ARNO-associated pathways and hence further understand ARNO function in SFs, naive and IL-1β-stimulated cells, the latter treated with and without ARNO siRNA, were subjected to RNAseq analysis. Principal Component Analysis identified that the three groups displayed distinct transcriptome profiles (**Figure 5A**) and so we therefore compared the significant differential gene expression [DE fold change > 4, adjp <0.01] existing amongst the three experimental groups (**Figure 5B**) to identify distinct transcriptomic signatures associated with each experimental group. Firstly, we wanted to identify which IL-1β-induced genes were suppressed by ARNO siRNA. We applied K-means clustering to all DE genes to find the genes that were up-regulated in IL-1β-stimulated, but not in ARNO siRNA IL-1β stimulated cells. A list of 122 genes was generated, of which 72 (**Supplementary Table 1**) were found to be functionally connected when subjected to String Protein-Protein Interaction Networks Functional Enrichment Analysis (41) (**Figure 5C**): the two most over-represented pathways were 1) CXC chemokines from the IL-8 superfamily, responsible for neutrophil and monocyte recruitment and 2) pro-inflammatory cytokines with crucial roles in joint inflammation, such as IL-6, CSF2, CSF3, IL-23 or TNFSF11 (RANKL). Interestingly, we found a significantly enriched number of cytokines induced downstream of JAK/STAT signalling (CSF2, CSF3, IL-6, IL-23a, LIF, TSLP), in line with the observed STAT3 activation by ARNO (**Figure 4**). Next, we used again the K-means approach to search for IL-1β-induced genes whose expression was enhanced when ARNO expression was silenced and this identified 94 genes, 32 functionally related (**Figure 5D**). Interestingly, this group included chemokine pathways (CCR3, CXCL13, CCL12, CCL3 and CCL4) and genes involved in immune signalling (JAK2, GBP2), although no inflammatory cytokines were present.

Overall, these results indicated that ARNO modulates IL-1β-driven inflammatory response rather than being essential for these functions. To examine the functional implications of such modulation, we directly compared IL-1β-stimulated SFs with those treated with IL-1β and ARNO siRNA SFs (**Figure 5E**), finding 384 up-regulated and 434 down-regulated genes [DE fold change > 2, adjp <0.01] (**Supplementary Table 2**). In addition to the distinct cytokine and chemokine signature, this analysis revealed a significant modulation of genes involved in cell migration and cytoskeleton organization, such as Arc or cadherins (Cdh1, Cdh5) (42–44) as well as genes that can activate ARFs (Arap2, Cytip and Mcoln2) (45–47). Intriguingly, ARNO silencing also modified the expression of genes involved in the biosynthesis of sialylated glycoproteins (Cmah, ST3Gal5, ST3Gal6, ST6Gal1), a pathway that we have recently shown to control SF activation in inflammatory arthritis (22). This DE gene list was also investigated for pathway enrichment using GO-term and KEGG databases (**Figure 5F**) and, although signalling pathways are generally regulated by post-translational modifications, our RNAseq data suggest that MAPK and RAS signaling pathways were affected by ARNO knock-down, with up- and down-regulation of pathway elements being indicated, perhaps explaining the observed differences in cytokine receptor pathways. Critically, focal adhesion and JAK-STAT pathways were specifically down-regulated in ARNO siRNA SFs, in agreement with our functional results, whilst GO-term analysis also confirmed the role of ARNO in cell adhesion and migration in these cells.

### Dual Role of ARNO in the Regulation of Pathogenic Responses to IL-1β

**Figure 6:**
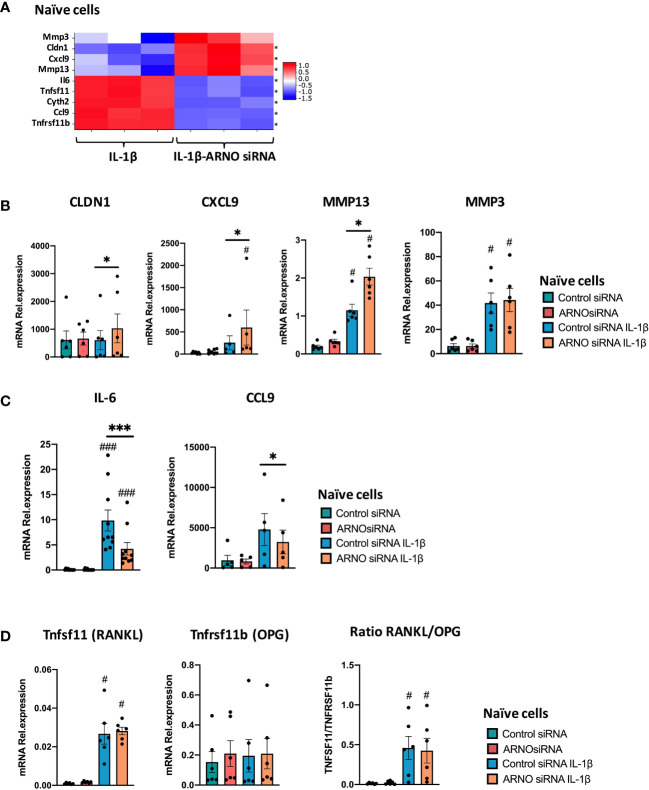
ARNO knock-down remodels the type of IL-1β-mediated inflammatory response. **(A)** mRNA expression as detected by RNA-Seq from **Figure 5** for ARNO (Cyth2), IL-6, CXCL9, CCL9, CLDN1, MMP3, MMP13, TNFRSF11b and TNFSF11. Differentially expressed genes with a adjp < 0.01 are shown by *. **(B–D)** RNA was isolated from unstimulated and IL-1β-stimulated naïve SFs upon Allstars/ARNO siRNA transfection. Relative mRNA expression of genes shown in **(A)** was evaluated by RT-qPCR using the ΔΔC_t_ method and actin as housekeeping gene. **(B)** CLDN1, Cxcl9, MMP13, MMP3, **(C)** IL-6, Ccl9, and **(D)** TNFRSF11b and TNFSF11 mRNA relative expression. For **(B–D)**, each dot represents one independent experiment (analysed in triplicate), error bars represent SEM (n≥5), *p < 0.05, ***p < 0.001 versus respective siRNA control, ^#^p < 0.05, ^###^p < 0.001 versus unstimulated control, statistical significance was evaluated by the Mann-Whitney test.

Our transcriptomic data suggest that ARNO is involved in regulating SF responses to IL-1β, being essential for IL-6 production and for directing responses towards specific chemokines. To further explore and confirm this, we selected a representative set of genes associated with inflammatory joint disease, whose expression was either significantly suppressed or enhanced by ARNO knock-down in the RNASeq dataset: specifically, we selected 4 down-regulated genes, IL-6, Ccl9, Tnfsf11 and Tnfrsf11b, and 4 up-regulated genes, MMP3, MMP13, CLDN1 and Cxcl9 (**Figure 6A**) and validated their expression status by qRT-PCR in independent SF cultures. Whilst the cytokines, chemokines and MMPs were chosen for their pro-inflammatory and matrix remodelling roles, CLDN1 was selected as a tight junction transmembrane protein involved in cell-cell adhesion (48). In addition, TNFSF11 (TNF Superfamily Member 11 also known as Receptor activator of nuclear factor kappa-B ligand, RANKL) is the major osteoclastogenic factor and TNFRSF11B (TNF Receptor Superfamily Member 11b, or Osteoprotegerin, OPG) acts as decoy receptor for RANKL and thus these genes are critical to the regulation of bone damage (49). Gene expression was evaluated by qRT-PCR analysis in independent experiments, using naïve murine SFs transfected with ARNO siRNA (or negative control Allstars siRNA) upon IL-1β stimulation as before. Corroborating our RNAseq experiment (**Figure 6A**), CLDN1, CXCL9 and Mmp13 expression was found to be up-regulated, although levels of Mmp3 were unaffected in ARNO knocked-down cells after IL-1β stimulation (**Figure 6B**). Levels of IL-6 and Ccl9 mRNA induced upon IL-1β activation were also reduced when ARNO was silenced (**Figure 6C**), although we did not observe any significant change in the expression of RANKL and OPG mRNA (**Figure 6D**).

**Figure 7:**
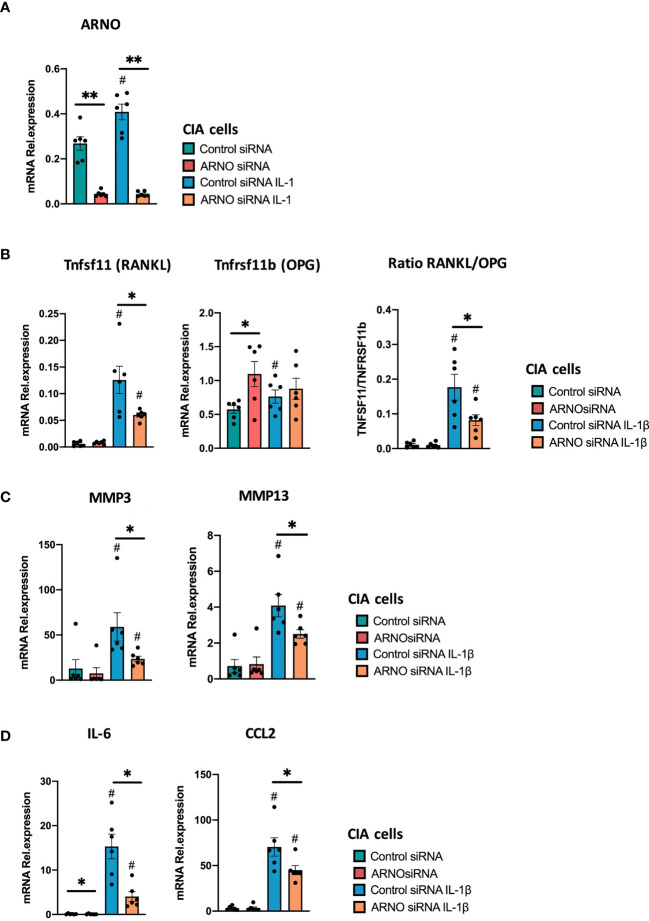
ARNO knock-down decreases IL-1β-dependent responses of CIA SFs. SFs were expanded from mice undergoing Collagen-Induced Arthritis, and RNA was isolated from unstimulated and IL-1β-stimulated cells upon ARNO knock-down by siRNA or Allstar treatment. ARNO **(A)** TNFRSF11b and TNFSF11 **(B)**, MMP3, MMP13 **(C)** and IL-6, CCL2 **(D)** relative mRNA expression was evaluated by RT-qPCR using the ΔΔC_t_ method and actin as housekeeping gene. Each dot represents one independent experiment (analysed in triplicate), error bars represent SEM, *p < 0.05, **p < 0.01 versus respective siRNA control, ^#^p < 0.05 versus unstimulated control, statistical significance was evaluated by the Mann-Whitney test.

Regulation of the RANKL/OPG axis by SFs has been shown to depend on the combined effects of inflammatory cytokines (50). Therefore, we repeated the experiment using fibroblasts from arthritic CIA mice, which have become epigenetically modified *in vivo* as a result of their chronic exposure to multiple cytokines and proinflammatory mediators (38, 51). Reflecting their imprinted pathogenic status, such CIA SFs showed a stronger induction of ARNO expression than naïve cells in response to IL-1β. Thus, whilst IL-1β induced 139% expression of ARNO in naïve cells (**Figure 1C**), CIA cells showed an 174% increase (**Figure 7A**). Importantly, ARNO siRNA transfection was equally effective at reducing ARNO mRNA expression (85.9% ± 0.05 reduction) in CIA cells (**Figure 7A**). Interestingly, RANKL/OPG balance was now dysregulated upon ARNO knock-down (**Figure 7B**), contrary to the results observed in naïve SFs (**Figure 6D**). Expression of RANKL after IL-1β stimulation was substantially reduced in CIA SFs, whilst that of OPG tended to be upregulated when ARNO expression was knocked-down and the differential regulation of these genes was highlighted by analysis of the ratio of their counter-regulatory expression (**Figure 7B**). These data suggest that ARNO-dependent regulation of gene expression differs in Naïve (**Figure 6**) and CIA SFs (**Figure 7**), with CIA SFs apparently more susceptible to adopting an inflammatory phenotype in response to the joint inflammatory mediator IL-1β. Providing further support for this idea, ARNO only promoted MMP13 expression in naïve SFs (**Figure 6B**) but inhibited it in CIA SFs (**Figure 7C**). Yet, ARNO siRNA treated CIA SFs show a significantly reduced ability to produce IL-6 and Ccl2 in CIA SFs (**Figure 7D**), similarly to naïve SFs.

## Discussion

In this study, we report that ARNO expression regulates two branches of SFs-dependent pathophysiology: migration and cytokine secretion. This is the first time that ARNO has been linked to synovial fibroblast biology, where we describe it not only as a regulator of cytoskeleton reorganization and cell migration, but also as a key player in adjusting local inflammatory responses. Reflecting this dual role, several cellular responses were dysregulated when ARNO expression was silenced, such as JAK-STAT signalling pathways, formation of focal adhesions, cell adhesion and secretory pathways, the latter of which is consistent with the known role of ARNO in other cell types. However, ARNO acting as a regulator of SF inflammatory pathways is a novel result that reveals a unique role for this cytohesin in synovial tissue. The role and mechanisms of ARNO-ARF6 in cell motility, cell adhesion and intracellular trafficking are well-established, but recent reports are now demonstrating its contribution in the regulation of inflammatory pathways, supporting our results in the context of synovitis. For example, ARF6 is required for MyD88-independent activation of TLR4 (52) and ARF6^-/-^ macrophages produced less inflammatory factors in a model of asthma (53) and consistent with the latter finding, ARNO, has been reported as a modulator of inflammatory responses in macrophages (20). Moreover, ARNO^-/-^ mice showed a reduced eosinophilic inflammation and IL-5 expression in an OVA-induced model of rhinitis (54). Finally, ARF6 regulates production of superoxide in neutrophils, as well as degranulation and their chemotaxis, polarization and adhesion (55–57). Nevertheless, these observations are still rare compared to those relating to its regulation of cytoskeletal functions, particularly protein trafficking, cell migration or recycling of membrane components and thus, we are only starting to understand how ARNO modulates inflammation in different cell types, including immune and non-immune cells.

Therefore, our results offer a new perspective for ARNO function, suggesting that it could be an integrator of migratory and SF-dependent inflammatory pathways. In this regard, it is worth remembering that cytoskeleton dynamics connect cellular responses with physical and chemical signals from the surrounding environment. For example, the RhoA/ROCK pathway regulates TNF-α-induced apoptosis through vimentin fiber remodelling (58), and vimentin regulates formation of focal adhesions and fibroblast proliferation and TGF-β1 signaling during wound healing (59). An interesting hypothesis would be that changes induced by IL-1β-ARNO-ARF6 in focal adhesions and cytoskeletal organisation equip SFs with the signalling pathways to sense and respond to the new mechanical and physical properties of the synovial space during infections or exposure to local stressors. ARNO could therefore be essential to maintain and fine-tune these inflammatory responses, but a prolonged exposure to environmental changes, like the increased tissue stiffness in arthritis, could dysregulate this mechanism, leading to the pathological migratory/inflammatory SF phenotype characteristic of RA. This is in line with our results, since ARNO bridges the two main pathological mechanisms observed in chronic joint disease, invasiveness and inflammation, likely a process that involves ARF6 activation. Nevertheless, further work is now needed to explore whether the IL-1β-ARNO-ARF6 axis can be an actual molecular sensor of inflammatory changes in local SFs, and which may offer new immunoregulatory checkpoints.

We found that inhibition of ARNO leads to inhibition of cytokine production upon IL-1β stimulation, including IL-6 and CCL2. Interestingly, this effect was greatly enhanced in SFs expanded from mice undergoing experimental Collagen-Induced Arthritis. This can be a consequence of the SFs epigenetically rewiring, by which cells adopt an inflammatory phenotype that is maintained *in vivo* and *ex vivo* (38, 60). Reflecting this transformation, ARNO appeared to be involved in MMP3 production only in CIA SFs, but not in healthy cells. This might be echoing the distinct activation of these SFs *in vivo*, and expanding the cellular functions for ARNO. Since MMP3 is a key enzyme in remodelling the matrix in the synovium, our results suggest again that ARNO is playing a differential role in sensing firstly, and responding secondly, to fluctuations in the synovial space. Furthermore, additional molecules may play a role in ARNO-dependent inflammatory migration, such as CXCL1/KC or CXCL2/MIP-2, functional homologues to the human IL-8, which are also down-regulated in ARNO knocked-down cells. The ARNO-ARF6 axis modulates Phosphatidylinositol-4-phosphate 5-kinases (PIP5Ks) and phospholipase D (PLD) to generate phosphoinositides and phospholipids (61, 62). Interestingly, these lipids are important in cytoskeleton remodelling and cell migration, offering potential links between IL-1β-ARNO-PIP5Ks/PLD pathways and SF activation. A better understanding of ARNO-mediated inflammatory pathways might facilitate identification of more selective therapeutics, since intervention of ARNO-dependent cytokine production could be a highly selective way to target SFs in joint inflammatory disease, in contrast to current drugs that are systemic immunosuppressors. To advance this, studies about ARNO function in fibroblasts from other anatomical locations are needed.

Other cytokines can also modulate ARNO expression. We tested IL-17, TNF and IL-1β, but only IL-1β influenced ARNO expression. This is in agreement with previous observations in endothelial cells, where ARNO-dependent maintenance of cell barrier permeability is also linked to IL-1β signalling (21). Because SFs also form a cellular barrier that separates the intra-articular space from the rest of the synovium, we cannot rule out that early activation of SFs by |L-1β in preclinical disease stages disrupts SF cell barrier formation, to allow leaking of inflammatory factors in the joint and initiation of inflammatory cascades. Those responses are likely to have a high degree of variability in the context of human inflammatory disease and could correlate with disease phenotypes that are driven by very specific sets of cytokines, like IL-17 or IL-1β (27). Whether ARNO-signalling can offer tailored therapies for specific inflammatory pathotypes should be addressed in future translational studies. A lower ratio of TNFSF11/TNFRSF11b was also found in ARNO knock-down pathogenic SFs compared with cells from heathy cells, suggesting that ARNO can induce bone damage in specific disease phenotypes, such as those driven by IL-1β. In this regard, analysis of the public repository Pathobiology of Early Arthritis Cohort (PEAC) RNA-Seq Data (https://peac.hpc.qmul.ac.uk) (63) shows that ARNO is up-regulated in SFs from leukocyte-rich RA patients, compared with leukocyte-poor RA phenotypes and cells from less inflammatory osteoarthritis (11). However, the physiological function and pathological mechanisms of this upregulation have not been studied. PEAC also shows that ARNO is highly expressed in CD34+ sub-lining SFs, a fibroblast subset characterized by secretion of large amounts of inflammatory cytokines (10). Notably, inhibition of cytohesins substantially reduces infiltration of inflammatory cells in the joint of animals undergoing experimental arthritis by affecting the endothelial barrier permeability (21), although the direct role of ARNO on SF-driven inflammation was not addressed. One of the limitations of our study was the use of an *ex vivo* model of SFs. Expanded SFs present a cell phenotype reminiscent of CD90+ sublining fibroblasts, but translation of our findings to human disease will require further studies in specific SFs subsets.

To understand the molecular mechanisms by which ARNO regulated cytokine production, we studied the effect of Cpd188, a selective STAT3 inhibitor. STAT3 has been associated with inflammatory responses (64, 65) and we found that ARNO knock down inhibits STAT3 phosphorylation and JAK/STAT signaling pathways. Cpd188 weakened SF migration, like ARNO silencing, but cytokine secretion was unaffected, perhaps suggesting that some additional STAT3 regulators are required for ARNO to modulate cytokine secretion or a completely different pathway(s). One candidate could be Rac1, an up-stream component of STAT3 signaling pathway and down-stream regulator of ARNO, whose activation promotes cellular secretion of IL-6 *via* the NF-κB-IL-6 axis (66–68). Furthermore, our preliminary data showing activation of STAT1 in CIA SFs suggest coupling to STAT1 in conjunction with STAT3 signalling could explain the differential effects of ARNO in healthy and CIA SFs. Interestingly, simultaneous activation of NF-κB and STAT3 was found to enhance the inflammatory responses of non-immune cells (69) whilst an IL-1β activated ERK/STAT1 signaling pathway was proposed to promote NF−κB−mediated cytokine secretion in rat synovial fibroblasts (70). Therefore, ARNO could modulate SF-dependent inflammation by affecting the balance of STAT1/STAT3-NFκB pathways, a hypothesis we are currently investigating. Alternatively, the effects of ARNO-signalling on other regulators of NF-κB-dependent cytokine expression are also being investigated, including that of the negative regulator SOCS3 (Suppressor Of Cytokine Signaling 3). Thus, a better understanding of the role of ARNO in regulating these signals both in naïve and arthrititic SFs may lead to more efficient clinical intervention of stromal cells in RA.

Overall, we report here that i) ARNO is necessary for reorganisation of focal adhesions and migration of synovial fibroblasts and ii) a novel role of ARNO in modulating the inflammatory response of SFs in response to IL-1β. This represents a direct connection between the pathophysiological migration of inflammatory fibroblasts and their ability to initiate and modulate local immune responses. Interestingly, ARNO did not act as a switch for inflammation, but rather modulated the type of inflammatory signals released by SFs. This role for ARNO may offer new options for modulating synovial fibroblast-mediated inflammation, cartilage and bone damage in chronic inflammatory joint diseases, such as RA, without severely compromising systemic immune responses.

## Data Availability Statement

The data presented in this study are deposited in NCBI's Gene Expression Omnibus and are accessible through GEO Series accession number GSE192488.

## Ethics Statement

The animal study was reviewed and approved by Ethics Review Board of the University of Glasgow.

## Author Contributions

MP conceived and oversaw the project, interpreted the results, and wrote the manuscript with feedback from all authors. CC contributed to data analysis and bioinformatic analysis. YW performed the experiments and contributed to design of the experiments, data analysis and manuscript writing. MH contributed to experimental design, data analysis and manuscript writing. All authors contributed to the article and approved the submitted version.

## Funding

The work was funded by a Career Development award to MP from Versus Arthritis (21221), a China Scholarship Council (CSC) PhD scholarship awarded to YW and a YLSY Turkish Ministry of National Education Study Abroad Programme awarded to CC.

## Conflict of Interest

The authors declare that the research was conducted in the absence of any commercial or financial relationships that could be construed as a potential conflict of interest.

## Publisher’s Note

All claims expressed in this article are solely those of the authors and do not necessarily represent those of their affiliated organizations, or those of the publisher, the editors and the reviewers. Any product that may be evaluated in this article, or claim that may be made by its manufacturer, is not guaranteed or endorsed by the publisher.

## Supplementary Material

Supplementary Figure 1 ARNO silencing does not affect Erk or c-Jun activation in CIA SFs. Representative western blots of ERK **(A)** and c-jun **(B)** expressed in CIA SFs treated with Allstars (control) and ARNO siRNA followed by IL-1β treatment (10ng/ml) at indicated times. Anti-pERK1/2, ERK1/2, p-c-jun^ser63^, c-jun and GAPDH antibodies were used. Graphs show the relative quantification of phosphorylated ERK1/2 and c-jun, calculated as p-ERK44/ERK44, p-ERK42/ERK42 and p-c-jun/c-jun band intensity ratios. Change in total protein expression were calculated as ERK44/GAPDH, ERK42/GAPDH and c-jun/GAPDH ratios. Each dot represents one independent experiment, error bars represent SEM (n=3 experiments for ERK1/2, n=2 experiments for c-jun).
