# Supplementary figures and images for: Cytohesin-2/ARNO: A Novel Bridge Between Cell Migration and Immunoregulation in Synovial Fibroblasts
Source: Front Immunol. 2022 Jan 12;12:809896. doi: 10.3389/fimmu.2021.809896 (PMC8790574; doi:10.3389/fimmu.2021.809896)

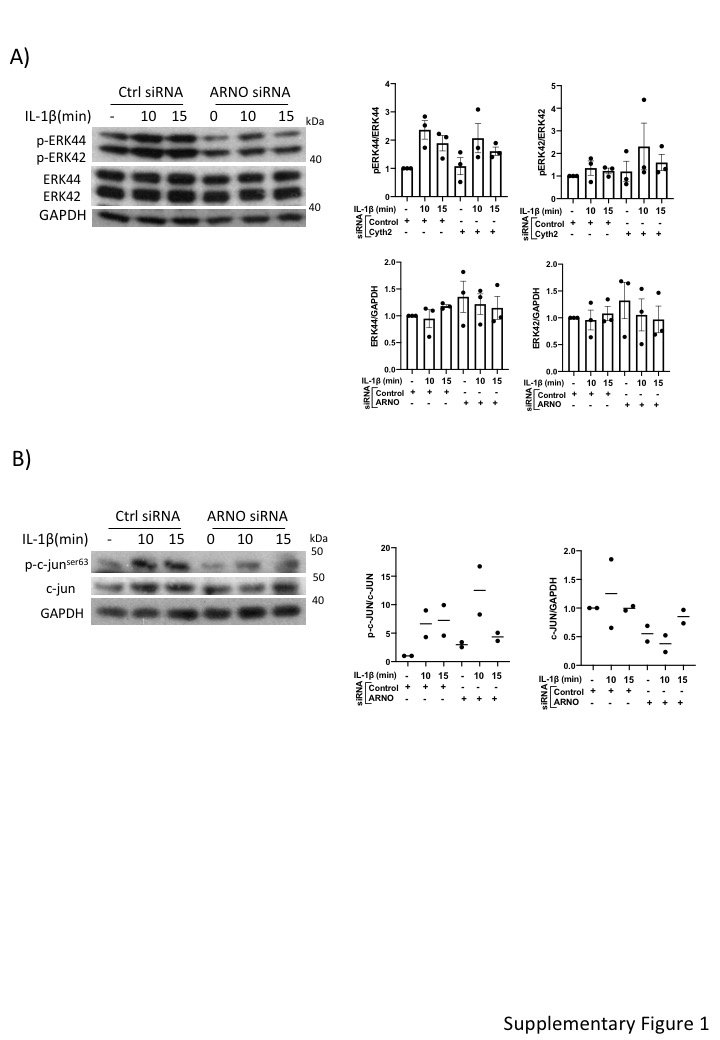

Supplement: Supplementary Figure 1 — ARNO silencing does not affect Erk or c-Jun activation in CIA SFs. Representative western blots of ERK (A) and c-jun (B) expressed in CIA SFs treated with Allstars (control) and ARNO siRNA followed by IL-1β treatment (10ng/ml) at indicated times. Anti-pERK1/2, ERK1/2, p-c-junser63, c-jun and GAPDH antibodies were used. Graphs show the relative quantification of phosphorylated ERK1/2 and c-jun, calculated as p-ERK44/ERK44, p-ERK42/ERK42 and p-c-jun/c-jun band intensity ratios. Change in total protein expression were calculated as ERK44/GAPDH, ERK42/GAPDH and c-jun/GAPDH ratios. Each dot represents one independent experiment, error bars represent SEM (n=3 experiments for ERK1/2, n=2 experiments for c-jun). [file Image_1.jpeg]
